# Case Report: Extremely Early Detection of Preclinical Magnetic Resonance Imaging Abnormality in Creutzfeldt–Jakob Disease With the V180I Mutation

**DOI:** 10.3389/fneur.2021.751750

**Published:** 2021-10-07

**Authors:** Ryuichi Koizumi, Naohisa Ueda, Atsushi Mugita, Katsuo Kimura, Hitaru Kishida, Fumiaki Tanaka

**Affiliations:** ^1^Department of Neurology, Yokohama City University Medical Center, Yokohama, Japan; ^2^Department of Neurology and Stroke Medicine, Yokohama City University Graduate School of Medicine, Yokohama, Japan

**Keywords:** Creutzfeldt–Jakob disease, diffusion-weighted image, magnetic resonance imaging, V180I mutation, early diagnostic marker

## Abstract

The diagnosis of presymptomatic Creutzfeldt–Jakob disease (CJD) is challenging. The levels of total tau protein, 14-3-3 protein, and protease-resistant isoform of prion protein (PrP^res^) in the cerebrospinal fluid; periodic sharp wave complexes on electroencephalography; and diffusion-weighted imaging (DWI) of brain magnetic resonance imaging (MRI) have all been used to diagnose symptomatic CJD, but none of these markers have been established in the diagnosis of presymptomatic CJD. Here, we report a case of genetic CJD with the V180I mutation in which a small punctate cortical hyperintensity was detected on DWI 6 months before symptom onset and 9 months before diagnosis. Presymptomatic CJD is currently impossible to diagnose because of the lack of established early diagnostic markers. However, since MRI is increasingly used in daily clinical practice, the chance detection of such DWI abnormalities would have important implications in terms of providing a clue to examine a highly specific early diagnostic marker to be developed in the future for CJD. This will allow presymptomatic intervention by disease-modifying therapy in the near future.

## Introduction

Creutzfeldt–Jakob disease (CJD) is a transmissible spongiform encephalopathy caused by the spread of pathological protease-resistant isoform of prion protein (PrP^res^) in the central nervous system. It is clinically characterized by rapidly progressing behavioral changes; psychiatric symptoms; dementia; myoclonus; cerebellar, pyramidal, and extrapyramidal signs; and visual disturbances and is invariably fatal (1). Diagnostic markers for CJD have been well-established and include total tau (t-tau) protein, 14-3-3 protein, and PrP^res^ detected by real-time quaking-induced conversion (RT-QuIC) in the cerebrospinal fluid (CSF) as well as periodic sharp wave complexes (PSWCs) on electroencephalography (EEG) and cortical and/or basal ganglia hyperintensity on diffusion-weighted imaging (DWI) of brain magnetic resonance imaging (MRI) (1).

However, the earliest diagnostic marker for CJD remains controversial. Satoh et al. (2) reported that CSF t-tau was a more sensitive marker than CSF 14-3-3 protein and DWI findings. Meanwhile, Shiga et al. (3) proposed that DWI is more sensitive and useful than the 14-3-3 protein and PSWCs. In contrast, the recently developed RT-QuIC assay on olfactory mucosa brushings was highly sensitive, according to the report by Zanusso et al. (4). Here, we report a case of genetic CJD with V180I mutation, in which a DWI abnormality was detected in the early stages of the disease.

## Case Presentation

**Figure 1:**
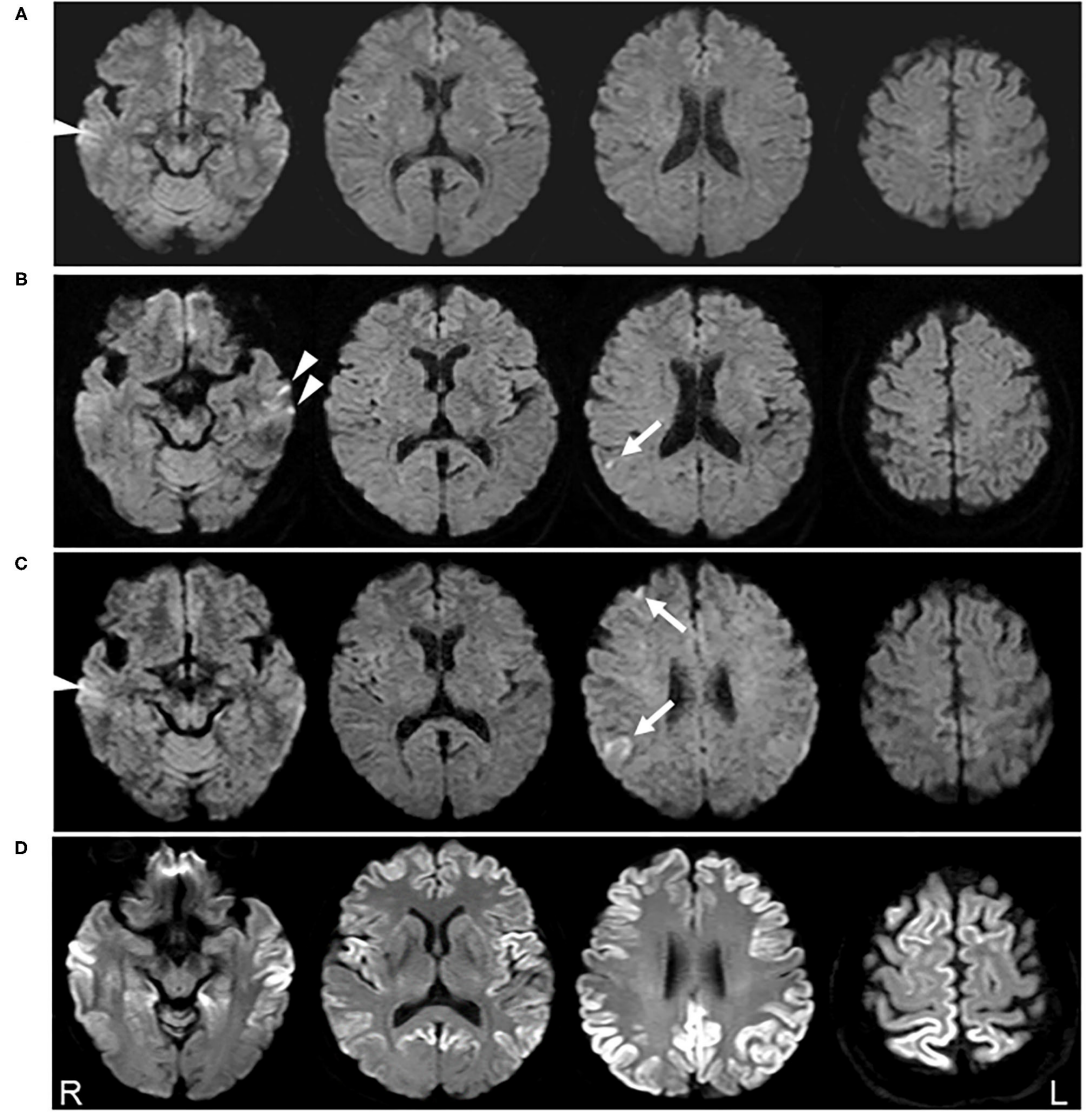
Serial diffusion-weighted images (DWI) of brain magnetic resonance imaging (MRI). No abnormalities were detected on DWI obtained 5 years prior to the clinical onset **(A)**. DWI acquired 6 months before clinical onset shows a very small hyperintensity in the right parietal cortex (arrow) **(B)**. DWI obtained 2 months before clinical onset reveals a slight increase in the abnormal lesion in the right parietal cortex and new lesions in the right frontal lobe (arrow) **(C)**. DWI obtained 3 months after clinical onset demonstrates the expansion of the hyperintense region to a broad range of the cerebral cortex **(D)**. Arrowheads shown in **(A–C)** possibly represent the artifacts in the temporal lobe cortex.

A 64-year-old Japanese woman with a history of uterine myoma developed an acute headache and visited a local hospital. She had no family members with any neurological disorders. On examination, she showed no neurological deficits, and cognitive function was normal. Brain MRI was performed to exclude intracranial tumors or strokes. Although no abnormalities were detected on MRI performed due to a slight headache 5 years prior (Figure 1A), DWI demonstrated very small hyperintensity in the right parietal cortex (Figure 1B, arrow). Apparent diffusion coefficient (ADC) images were not acquired. She was diagnosed with acute cerebral infarction, and aspirin intake was initiated. Her headache disappeared shortly thereafter.

Follow-up MRI performed 4 months later revealed new lesions in the right frontal lobe, in addition to a slight enlargement of the previous lesion (Figure 1C, arrows). Recurrence of cerebral infarction was suspected by the physician. Because the patient was asymptomatic and the DWI lesions were still small and restricted to the cortices, there was no strong reason to suspect other diseases affecting the cerebral cortex. Accordingly, no additional treatment was administered.

Two months later, she developed a mild action tremor in her extremities, which gradually deteriorated. Three months later, she was admitted to our institution for rapidly progressive dementia and gait disturbance. Neurological examination revealed a decline in cognitive function [Mini-Mental State Examination (MMSE), 16/30], ideomotor apraxia in the right hand, cortical sensory dysfunction in the bilateral hands, action tremor in the extremities, and gait disturbance. Myoclonus was not observed. DWI demonstrated expansion of the hyperintense area to a broad range of the cerebral cortex (Figure 1D). CSF analysis revealed a *t*-tau protein level of more than 2,200 pg/ml and positivity for the 14-3-3 protein. The CSF RT-QuIC assay was negative for PrP^res^. EEG did not show any PSWCs. Prion protein gene analysis detected a point mutation (V180I), while a study of the polymorphism at codon 129 revealed Met/Val heterozygosity (129MV). A year later, her cognitive function further declined, and she became mute. However, she could still walk with partial assistance. Myoclonus was not observed during the follow-up period.

## Discussion

High signal abnormalities on DWI in the cortical regions and/or in the striatum are of significant diagnostic value for CJD. In our case of V180I CJD, a cortical DWI hyperintense change was successfully detected in the extremely early stage of the disease before the emergence of CJD symptoms (Figure 1B). To the best of our knowledge, abnormal DWI lesions have been detected in seven previously reported CJD cases before the onset of symptoms (Table 1) (5–11). Six of these were sporadic CJD (5, 7–11), and one was a case of genetic CJD with V180I mutation (6), as in our case. The duration between initial MRI detection and symptom onset was 2–16 months, consistent with that in our case where the initial MRI abnormality was detected 6 months before the onset of CJD symptoms (action tremor) and 9 months before the diagnosis.

**Table 1:** CJD cases with abnormal DWI hyperintensity before symptom onset.

**Case no**.	**Age/sex**	**Time from MRI to symptom onset (months)**	**Motive for MRI performance**	***t*-tau**	**14-3-3 protein**	**Rt-QuIC**	**PSWC on EEG**	**Subtype of CJD (mutation)**	**Codon 129 genotypes and protein types**	**References**
1	68/M	3	Patient's request	+	-	NE	NE	sCJD	MM1	(5)
2	68/F	3	Headache	NE	NE	NE	-	gCJD (V180I)	MV	(6)
3	65/F	12	Headache	NE	NE	NE	NE	sCJD	MM	(7)
4	74/F	12	Cervical tumor	NE	NE	NE	NE	sCJD	MM1	(8)
5	77/M	2	Dizziness	NE	NE	NE	NE	sCJD	MM1+2	(9)
6	67/F	12	Medical checkup	-	-	NE	-	sCJD	MM	(10)
7	64/M	16	Headache	-	-	-	-	sCJD	MV	(11)
This case	65/F	6	Headache	NE	NE	NE	-	gCJD (V180I)	MV	

*DWI, diffusion-weighted imaging; EEG, electroencephalography; gCJD, genetic Creutzfeldt–Jakob disease; MM, homozygosity for methionine; MRI, magnetic resonance imaging; MV, methionine/valine heterozygosity; NE, not examined; PSWC, periodic sharp wave complexes; RT-QuIC, real-time quaking-induced conversion; sCJD, sporadic Creutzfeldt–Jakob disease*.

However, hyperintense DWI lesions in the reported patients were already linear in shape or exhibited typical findings that were highly suspicious of CJD. In our initial DWI, it was difficult to determine whether the hyperintense lesion was due to CJD, because the lesion was punctate in appearance (Figure 1B). In fact, the patient was initially diagnosed with cerebral infarction. Interestingly, stroke-like onset of CJD has also been reported (12), and, accordingly, cerebral infarction is an important differential diagnosis in terms of radiological and clinical aspects. In addition to cerebral infarction, many differential diagnoses for cortical DWI features mimicking CJD have been reported, including hypoxic ischemic encephalopathy, hypoglycemia, autoimmune-mediated encephalopathy, encephalitis, status epilepticus, hyperammonemia, and mitochondrial disorders (13); differential diagnosis can therefore be difficult, especially in the early disease stage with no or non-specific symptoms.

Remarkably, asymptomatic DWI lesions were detected in two cases harboring a V180I mutation with 129MV, including our own (Table 1). In Japan, V180I is the most prevalent genetic cause of CJD and is generally characterized by slow progression (14). However, 129MV comprises only 8.4% of the normal population in Japan (15); therefore, the clinical features of V180I patients with 129MV are largely unknown, unlike those with 129MM. It may be reasonable to consider that the slowly progressive form of CJD may have a greater chance of detecting asymptomatic DWI abnormalities. As shown in Table 1; however, it is difficult to confirm this hypothesis and the effects of the polymorphism at codon 129 because well-known rapidly progressive forms with 129MM type 1 are also included.

Among the seven previously reported cases with positive DWI findings before symptom onset, t-tau protein levels in the CSF were also elevated (1,433 pg/ml) in one of three cases (Table 1), but a mildly elevated level (1,200–2,000 pg/ml) has also been observed in other neurological disorders, such as Alzheimer's disease. EEG and CSF analysis, such as for the 14-3-3 protein and RT-QuIC, remained normal in cases where data were available (Table 1). From these reports, CSF or EEG analysis has an inferior sensitivity for detecting asymptomatic CJD pathogenesis compared to DWI. In particular, CJD patients with the V180I mutation have low rates of positivity for t-tau protein, 14-3-3 protein, RT-QuIC, and PSWC, even after symptom onset (16). In addition, CSF analysis is rarely performed before CJD is suspected because of its invasiveness. In contrast, MRI scans are often performed even for mild neurological symptoms, such as headache and dizziness, at least in Japan; hence, there is a possibility of chance detection of extremely early MRI abnormalities in CJD, as in our case. However, caution should be exercised when interpreting DWI signals, especially in the frontal and temporal cortices (17). For example, when a very small hyperintensity was detected in the right parietal cortex, left temporal lesions were also suspected (Figure 1B, arrowheads). However, they could not be detected on follow-up MRI (Figure 1C). Furthermore, hyperintensity found in the right temporal cortex in Figure 1C (arrowhead) was also seen, even on MRI performed 5 years prior (Figure 1A, arrowhead). These findings suggest that temporal lobe hyperintensities observed in Figures 1A–C may be artifacts.

Although it might be extremely rare, chance detection of cortical small hyperintense DWI lesions without corresponding symptoms (Figures 1B,C, arrows) should be carefully followed up, considering the possible diagnosis of CJD. When we encounter such DWI findings, the currently available diagnostic markers for CJD are not very useful, as shown in Table 1. Hopefully, the use of highly specific and sensitive markers expected to be developed in the future will help diagnose asymptomatic CJD, which is important for early intervention in future disease-modifying therapy. In this setting, careful counseling and psychological support will be needed for asymptomatic patients, because even after disease-modifying therapy to delay the progression, CJD remains a lethal disease. This is also applicable to the family members of genetic CJD patients, even though CJD with V180I mutation mostly occurs as sporadic CJD and is rarely reported in family members (14).

There are several limitations in this report. First, ADC mapping was not performed during the course of the disease. ADC values are generally reported to decrease in patients with CJD, but not in the early stages (6). Therefore, ADC mapping may be useful to differentiate small cortical DWI hyperintensities observed in presymptomatic CJD from cerebral infarction. Without this information, we cannot completely exclude the etiologic possibility of small cerebral infarction (Figure 1B). Second, due to the rarity of presymptomatic DWI-positive CJD cases, we were not able to extract the clinical features of these cases, including the polymorphism at codon 129. Further accumulation of CJD cases with preclinical DWI abnormalities will solve this issue.

## Data Availability Statement

The original contributions presented in the study are included in the article/supplementary material, further inquiries can be directed to the corresponding author/s.

## Ethics Statement

Ethical review and approval was not required for the study on human participants in accordance with the local legislation and institutional requirements. Written informed consent for participation was not required for this study in accordance with the national legislation and the institutional requirements.

## Author Contributions

RK and NU performed case information collection, literature review, and drafted the manuscript. AM, KK, and HK contributed to the collection of case information and literature information statistics. FT performed the manuscript review and approved the final version. All authors contributed to the manuscript and approved the submitted version.

## Conflict of Interest

The authors declare that the research was conducted in the absence of any commercial or financial relationships that could be construed as a potential conflict of interest.

## Publisher's Note

All claims expressed in this article are solely those of the authors and do not necessarily represent those of their affiliated organizations, or those of the publisher, the editors and the reviewers. Any product that may be evaluated in this article, or claim that may be made by its manufacturer, is not guaranteed or endorsed by the publisher.
